# Host-feeding patterns of mosquito species in Germany

**DOI:** 10.1186/s13071-016-1597-z

**Published:** 2016-06-03

**Authors:** Jessica Börstler, Hanna Jöst, Rolf Garms, Andreas Krüger, Egbert Tannich, Norbert Becker, Jonas Schmidt-Chanasit, Renke Lühken

**Affiliations:** Bernhard Nocht Institute for Tropical Medicine, WHO Collaborating Centre for Arbovirus and Hemorrhagic Fever Reference and Research, Hamburg, Germany; German Centre for Infection Research (DZIF), partner site Hamburg-Luebeck-Borstel, Hamburg, Germany; Department of Tropical Medicine, Bundeswehr Hospital Hamburg, Hamburg, Germany; German Mosquito Control Association (KABS), Institute for Dipterology, Speyer, Germany; University of Heidelberg, Heidelberg, Germany

**Keywords:** Mosquito, Host species, Host-feeding pattern, Germany

## Abstract

**Background:**

Mosquito-borne pathogens are of growing importance in many countries of Europe including Germany. At the same time, the transmission cycles of most mosquito-borne pathogens (e.g. viruses or filarial parasites) are not completely understood. There is especially a lack of knowledge about the vector capacity of the different mosquito species, which is strongly influenced by their host-feeding patterns. While this kind of information is important to identify the relevant vector species, e.g. to direct efficient control measures, studies about the host-feeding patterns of mosquito species in Germany are scarce and outdated.

**Methods:**

Between 2012 and 2015, 775 blood-fed mosquito specimens were collected. Sampling was conducted with Heavy Duty Encephalitis Vector Survey traps, Biogents Sentinel traps, gravid traps, hand-held aspirators, sweep nets, and human-bait collection. The host species for each mosquito specimen was identified with polymerase chain reactions and subsequent Sanger sequencing of the cytochrome *b* gene.

**Results:**

A total of 32 host species were identified for 23 mosquito species, covering 21 mammalian species (including humans) and eleven bird species. Three mosquito species accounted for nearly three quarters of all collected blood-fed mosquitoes: *Aedes vexans* (363 specimens, 46.8 % of all mosquito specimens), *Culex pipiens pipiens* form *pipiens* (100, 12.9 %) and *Ochlerotatus cantans* (99, 12.8 %). Non-human mammals dominated the host species (572 specimens, 73.8 % of all mosquito specimens), followed by humans (152, 19.6 %) and birds (51, 6.6 %). The most common host species were roe deer (*Capreolus capreolus*; 258 mosquito specimens, 33.3 % of all mosquito specimens, 65 % of all mosquito species), humans (*Homo sapiens*; 152, 19.6 %, 90 %), cattle (*Bos taurus*; 101, 13.0 %, 60 %), and wild boar (*Sus scrofa*; 116, 15.0 %, 50 %). There were no statistically significant differences in the spatial-temporal host-feeding patterns of the three most common mosquito species.

**Conclusions:**

Although the collected blood-fed mosquito species had a strong overlap of host species, two different host-feeding groups were identified with mosquito species feeding on (i) non-human mammals and humans or (ii) birds, non-human mammals, and humans, which make them potential vectors of pathogens only between mammals or between mammals and birds, respectively. Due to the combination of their host-feeding patterns and wide distribution in Germany, *Cx. pipiens pipiens* form *pipiens* and *Cx. torrentium* are potentially most important vectors for pathogens transmitted from birds to humans and the species *Ae. vexans* for pathogens transmitted from non-human mammals to humans. Finally, the presented study indicated a much broader host range compared to the classifications found in the literature for some of the species, which highlights the need for studies on the host-feeding patterns of mosquitoes to further assess their vector capacity and the disease ecology in Europe.

**Electronic supplementary material:**

The online version of this article (doi:10.1186/s13071-016-1597-z) contains supplementary material, which is available to authorized users.

## Background

Mosquito-borne pathogens are of growing importance in many countries of Europe [1]. Due to intensified surveillance over the last seven years, different pathogens were also detected in mosquito specimens in Germany for the first time, including three different viruses (Sindbis virus (SINV) [2], Batai virus (BATV) [3] and Usutu virus (USUV) [4]), two filarial parasites (*Dirofilaria repens* [5, 6], *D. immitis* [6]), and three species of the *Borrelia burgdorferi* complex (*B. afzelii*, *B. bavariensis* and *B. garinii* [7]). However, the transmission cycles of these pathogens are hardly understood. There is especially a lack of knowledge about the vector capacity of the different mosquito species. Thereby, besides vector competence or spatial-temporal abundance, host-feeding patterns are an important information to identify potential vector species under natural conditions [8, 9], e.g. to direct efficient control measures [10].

Several studies identified the host species of mosquitoes [11, 12]. However, restricted by the available technologies, before 1996 [13], early works were mostly limited to a distinction of broad host groups rather than the identification of explicit host species, e.g. “bird” instead of “Common blackbird (*Turdus merula*)” [12]. However, mosquitoes are known to discriminate their hosts beyond the level of host groups [13] and e.g. DNA barcoding of engorged mosquito specimens is assumed to give a more specific insight into host-feeding patterns [12, 14].

In addition, only few studies evaluated the feeding behavior of European mosquitoes. Most of these studies focused on already known vector species, e.g. *Culex* spp. for West Nile virus (WNV) or avian malaria parasites [15, 16] or *Aedes albopictus* for a variety of pathogens [16], but only few studies collected data on a wide range of European mosquito species [17]. Information on the host-feeding patterns of mosquito species in Germany mostly rely on anecdotal behavioral observations [18, 19] or reviews of expert knowledge [20]. To our knowledge, no study evaluated the host-feeding patterns of mosquito species in Germany by the identification of blood content in the mosquito gut of specimens collected in the field.

Therefore, a molecular approach was utilized to (i) characterize the general host-feeding patterns of mosquitoes and their spatial-temporal variation; (ii) classify host-feeding groups to identify potential vector species; and (iii) estimate the potential transmission risk from birds to humans and from non-human mammals to humans for each mosquito species. These aims were achieved by the collection of blood-fed mosquitoes in 52 different study sites all over Germany using different trapping methods, which in combination allowed the collection of 20 different mosquito species. The results demonstrated, that many species had a broader host-species range than commonly assumed in the literature and a strong overlap for certain host-species (i.e. roe deer, humans, cattle and wild boar), indicating that host-feeding patterns and thereby the risk of pathogen transmission is probably more influenced by the host species availability than by species-specific host choices. However, at the same time, at least two distinct host-feeding groups were identified: (i) non-human mammals and humans; and (ii) birds, non-human mammals and humans, highlighting the mosquito species potential as vectors for pathogens between mammals and between mammals and birds, respectively. Due to the combination of host-feeding pattern and species abundance, *Cx. pipiens pipiens* form *pipiens* and *Cx. torrentium* are potentially important vectors for pathogens transmitted from birds to humans and *Ae. vexans* for pathogens transmitted from non-human mammals to humans.

## Methods

**Fig. 1:**
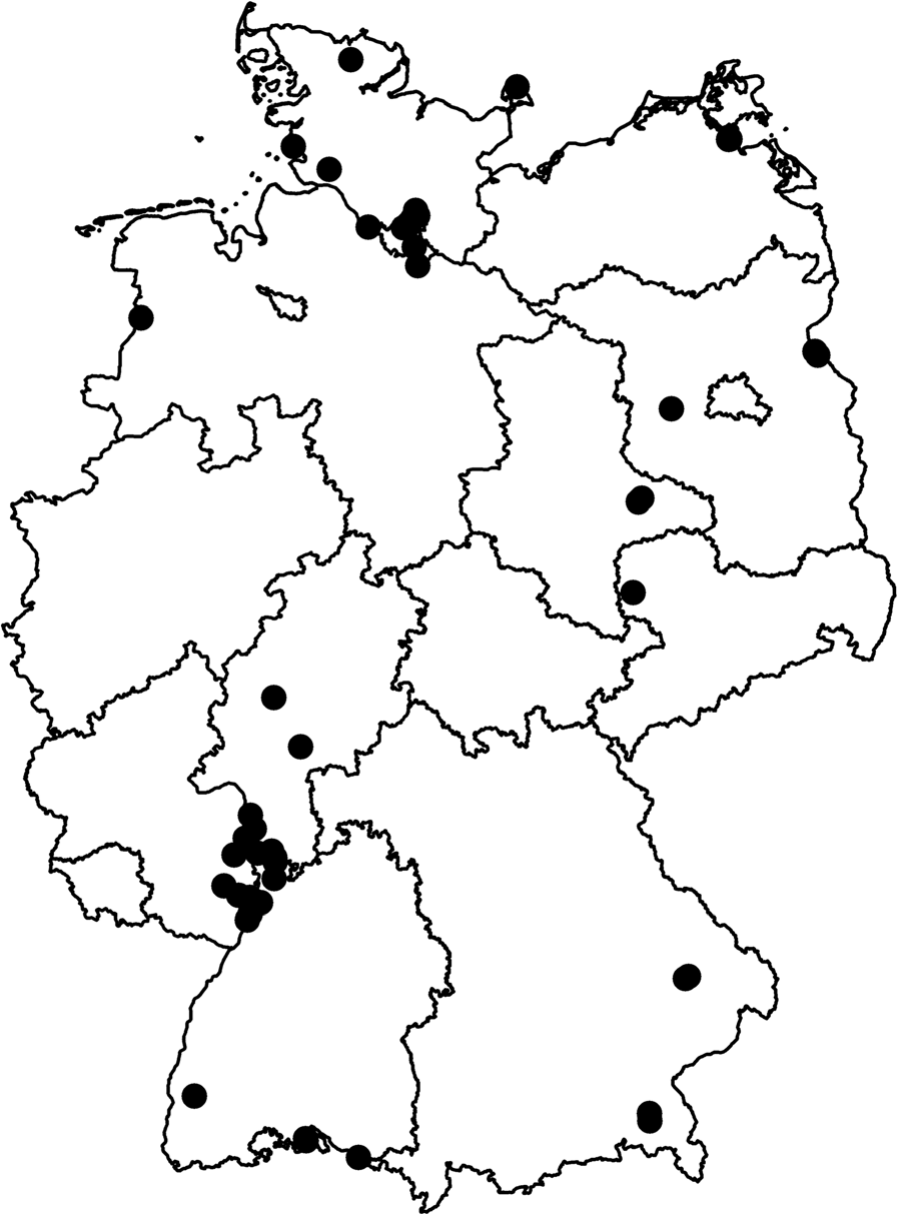
Trapping sites of the analysed blood-fed mosquitoes in Germany

Blood-fed mosquitoes were collected as by-catch within a nationwide mosquito and pathogen surveillance program at 52 trapping sites in Germany between 2012 and 2015 (Fig. 1, Additional file 1: Table S1). The dominating land use around the trapping sites could be characterised as “urban” (11 sites), “rural” (36 sites) or “natural” (5 sites). Sampling was conducted with Heavy Duty Encephalitis Vector Survey traps (EVS traps; BioQuip Products, Rancho Dominguez, California, USA; http://www.bioquip.com/) with CO_2_ from dry ice (601 mosquito specimens) or yeast (1 specimen); Biogents Sentinel traps (BioGents, Regensburg, Germany, http://www.biogents.com/) with CO_2_ from a gas cylinder (34 mosquito specimens) or without CO_2_ (3 specimens); gravid traps designed according to the CDC (Centers for Disease Control and Prevention) gravid trap model 1712 (John W. Hook Company, Gainesville, Florida, USA) with a hay infusion as oviposition attractant [21] (42 mosquito specimens); hand held aspirators (20 mosquito specimens); sweep nets (71 mosquito specimens); or human-bait collection (3 mosquito specimens). All mosquitoes were stored immediately on dry ice and transported to the laboratory. Completely engorged, partly blood-fed as well as females with nearly digested blood were used for further analysis. Each specimen was identified on chill tables using morphological characters [22, 23]. Morphologically identified *Cx. pipiens* (*sensulato*) (*s.l*.)/*torrentium* specimens were identified to the species level (*Cx. pipiens pipiens* form *pipiens*, *Cx. pipiens pipiens* form *molestus* and *Cx. torrentium*) using a molecular DNA typing assay [24]. This PCR assay did not work for ten specimens and therefore were summarized as *Cx. pipiens* (*s.l.*)/*torrentium*.

DNA isolation was performed from the whole mosquito body. Specimens were placed into 2 ml tubes and about 20 pieces of 2.0 mm zirconia beads (BioSpec Products, Bartlesville, USA) as well as 1 ml of cell culture medium (high-glucose Dulbecco’s modified Eagle’s medium; Sigma-Aldrich, St. Louis, MO, USA) were added. The homogenization was performed with a Tissuelyser LT (Qiagen, Hilden, Germany) for 2 min at 50 oscillations/s and 200 μl of the homogenate were used for DNA extraction, which was performed with KingFisher™ Flex Magnetic Particle Processor using MagMAX™ Pathogen ribonucleic acid/DNA Kit (both Thermo Fisher Scientific, Waltham, MA, USA). Polymerase chain reaction (PCR) amplification of mitochondrial cytochrome *b* gene was conducted with the primers presented by Kitano et al. [25]: L2513: 5′- GCC TGT TTA CCA AAA ACA TCA C-3′ and H2714: 5′-CTC CAT AGG GTC TTC TCG TCT T-3′ (~244 bp). As summarized by Schönenberger et al. [17], the host-species identification by PCR can be biased by the taxa-specific sensitivity of the different primer sets. Therefore, if the PCR with the primers by Kitano et al. [25] failed, i.e. no PCR amplicon was produced, an additional PCR with a second set of primers was used to increase the chance to gain PCR amplicons: L14841: 5′-CCA TCC AAC ATC TCA GCA TGA TGA AA-3′ and H15149: 5′-CCC TCA GAA TGA TAT TTG TCC TCA-3′ (~358 bp) [26]. PCR reactions were performed with the HotStarTaq Plus Master Mix Kit (Qiagen, Valencia, CA) according the following protocol: incubation at 95 °C for 5 min, 40 cycles at 94 °C for 30 s, 57 °C for 30 s, and 72 °C for 30 s. The PCR reaction was completed by incubation at 72 °C for 5 min. Amplicons were visualized by electrophoresis in a 2 % agarose gel with added Midori Green Advance (Biozym Biotech, Hessisch Oldendorf, Germany). Blood samples from humans (*Homo sapiens*), moose (*Alces alces*) or European blackbird (*Turdus merula*) were used as positive controls and distilled water (Ampuwa, Fresenius Kabi Deutschland GmbH, Bad Homburg, Germany) as negative control. Sanger sequencing was applied for all positive amplicons (LGC Genomics, Berlin, Germany), sequences pre-processed with Geneious® 7.1.9 and compared to sequences from the GenBank database (National Center for Biotechnology Information, http://blast.ncbi.nlm.nih.gov/Blast.cgi).

Data analysis was conducted with R [27]; the packages plyr [28] and reshape2 [29] were used for data manipulation and the packages ggplot2 [30], gridExtra [31] and ggdendro [32] for data visualization.

The general feeding patterns were evaluated with a null-model analysis. Thereby, the observed feeding pattern is compared to randomizations of the same dataset to test if the pattern is random (without host-specificity), shows a strong segregation of host species or aggregation around at least one host (see Chaves et al. [12] for further methodological details). This analysis was conducted separately for the complete dataset (host patterns between the mosquito species) and for the three most frequent mosquito species *Ae. vexans*, *Cx. pipiens pipiens* form *pipiens* and *Oc. cantans* (host patterns between the trapping sites). The analysis was performed with the R package EcoSimR [33] using the fixed-equiprobable algorithm and 5000 randomizations.

Furthermore, the impact of the surrounding land use and sampling period on the host-feeding patterns of the three most frequent mosquito species was analysed. For each trapping site, the proportion of aggregated land cover variables (urban = 111–142, rural = 211–244, natural = 311–423; Corine Land Cover 2006 raster data, http://blast.ncbi.nlm.nih.gov/Blast.cgi) in a 2500 m buffer was calculated. According to the dominance of one of the three aggregate land cover variables, the surrounding land use of the trapping sites was either classified as “urban” (5 sites), “rural” (28 sites) or “natural” (5 sites). Differences of the percentages of detected birds, non-human mammals or humans between the three land use classes were tested for significance using a Kruskal-Wallis test from the R package *stats* [28]. For the temporal analysis, the frequencies of detected birds, non-human mammals or humans were compared with Chi-square tests between two sampling periods [first half of the year (January to June) and the second half of the year (July to December)]. In addition, differences in the frequencies of detected mammalian or avian hosts among all pairs of used trapping methods were compared with Chi-square tests with adjusted *P*-values for multiple comparisons from the R package fifer [34].

A dendrogram of the presence-absence dataset of host-feeding groups (birds, non-human mammals or humans) for all mosquito species (normalized dataset) was produced based on Jaccard’s dissimilarity index and average agglomeration method with the R package *vegan* [35].

Finally, for each mosquito species, the transmission risk for pathogens from birds to humans (e.g. WNV or USUV) and from non-human mammals to humans (e.g. BATV) was estimated. The formulae in the publication by Muñoz et al. [15] [e.g. infection risk of birds = A (abundance of mosquitoes estimated as the mean number of females captured per trap/night) * Fa (fraction of blood meals taken from avian hosts) * Fa * Cv (measurement of the vector competence estimated as the proportion of bites from infected mosquitoes that transmit the virus)] was adapted and used without the parameter for vector competence (Cv), because vector competence data from other countries are probably not applicable for mosquito populations in Germany. For example, Leggewie et al. [36] identified geographical variation of WNV susceptibility even between different mosquito populations in the same country. Furthermore, the vector competence of most mosquito species in Germany was not studied yet. Therefore, two formulae were used to estimate the pathogen transmission risks to humans: (i) transmission risk from birds to humans = Ap * Fa * Fh; and (ii) transmission risk from non-human mammals to humans = Ap * Fm * Fh, with the parameters Ap (percentage abundance of mosquitoes), Fa (fraction of blood meals taken from avian hosts), Fh (fraction of blood meals taken from human hosts) and Fm (fraction of blood meals taken from non-human mammalian hosts). The information on the percentage abundance of mosquitoes (parameter Ap) over all trapping sites was derived from the complete dataset (approximately 350,000 specimens) of the nationwide mosquito and pathogen surveillance program from 2012 to 2015. *Culex pipiens pipiens* form *pipiens*, *Cx. pipiens pipiens* form *molestus* and *Cx. torrentium* were not differentiated within this dataset. Therefore, the percentage abundance information of *Cx. pipiens* (*s.l*.)/*torrentium* was used in the transmission risk calculations for *Cx. pipiens pipiens* form *pipiens* and *Cx. torrentium*.

## Results

**Fig. 2:**
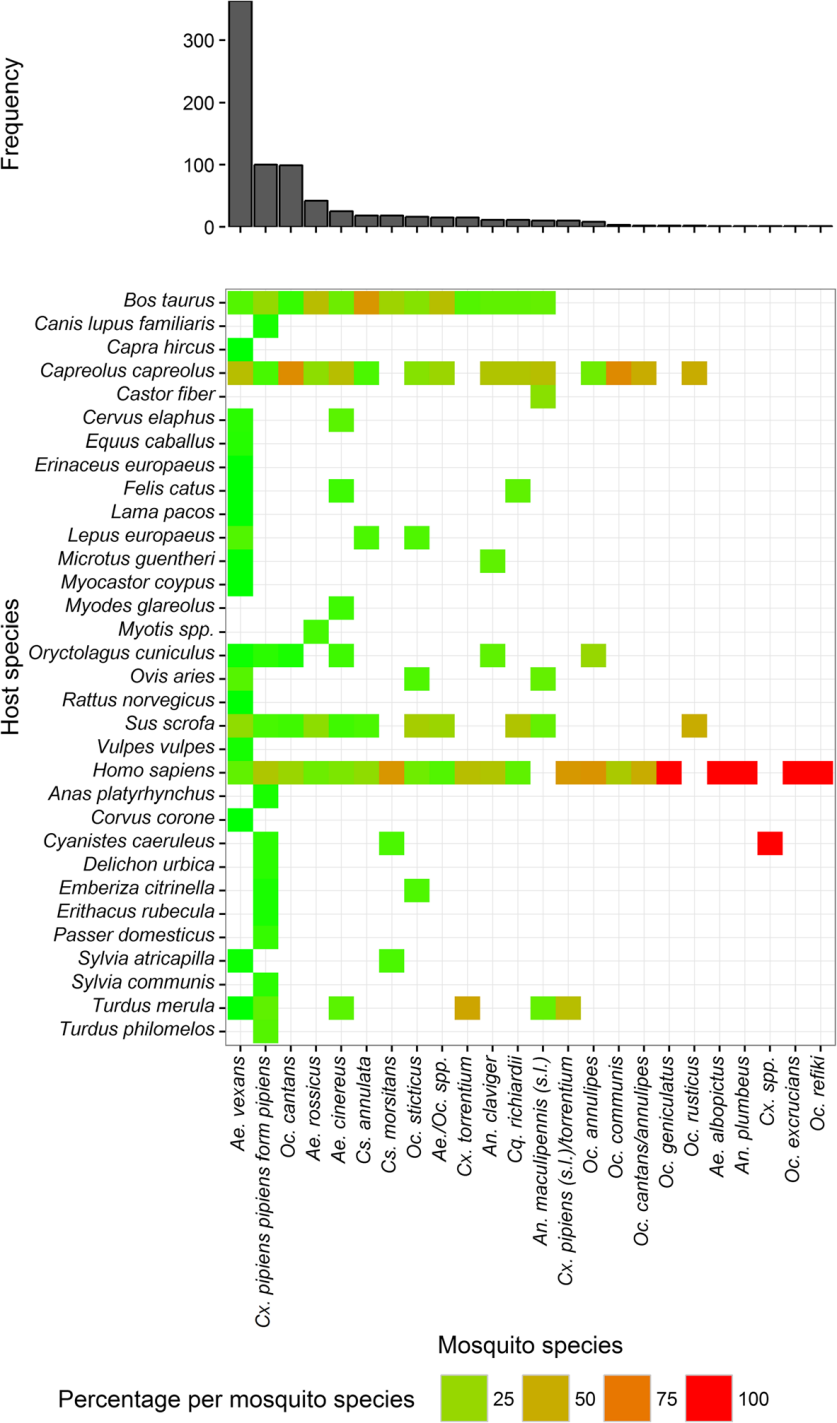
Frequency of each mosquito species and percentage of each detected host species per mosquito species

A total of 32 host species were determined from 775 blood-fed mosquitoes of 20 different mosquito species and four unspecified taxa (*Aedes/Ochlerotatus spp., Culex spp., Cx. pipiens *(*s.l.*)*/torrentium, Oc. cantans annulipes*) covering 21 mammalian species (including humans) and eleven bird species (Fig. 2, Tables 1 and 2). Three mosquito species accounted for nearly three quarters of all collected specimens: *Ae. vexans* (363 specimens, 46.8 % of all mosquito specimens), *Cx. pipiens pipiens* form *pipiens* (100, 12.9 %), and *Oc. cantans* (99, 12.8 %) (Table 1). All other species were trapped with considerably lower numbers (1–42 specimens). By far the highest number of host species was detected for *Ae. vexans* (21 species) and *Cx. pipiens pipiens* form *pipiens* (15 species), whereas only five host species were detected for the third most frequent mosquito species *Oc. cantans*.

**Table 1:** Frequency and percentage of each mosquito species with information on the frequency of detected host-feeding groups (birds, non-human mammals and humans) and number of detected host species

Mosquito species	No. of mosquito specimens	Percentage of all collected specimens	Birds	Non-human mammals	Humans	Host species
*Aedes albopictus*	1	0.1	0	0	1	1
*Aedes cinereus*	25	3.2	2	19	4	9
*Aedes rossicus*	42	5.4	0	37	5	5
*Aedes vexans*	363	46.8	4	325	34	21
*Aedes*/*Ochlerotatus* spp.	15	1.9	0	14	1	4
*Anopheles claviger*	11	1.4	0	7	4	5
*Anopheles maculipennis* (*s.l*.)	10	1.3	1	9	0	6
*Anopheles plumbeus*	1	0.1	0	0	1	1
*Coquillettidia richiardii*	11	1.4	0	10	1	5
*Culiseta annulata*	18	2.3	0	14	4	5
*Culiseta morsitans*	18	2.3	2	5	11	4
*Culex pipiens pipiens* form *pipiens*	100	12.9	28	37	35	15
(*Culex pipiens* (*s.l.*) /*torrentium*	10	1.3	4	0	6	2
*Culex* spp.	1	0.1	1	0	0	1
*Culex torrentium*	15	1.9	8	1	6	3
*Ochlerotatus annulipes*	8	1.0	0	3	5	3
*Ochlerotatus cantans*	99	12.8	0	73	26	5
*Ochlerotatus cantans*/*annulipes*	2	0.3	0	1	1	2
*Ochlerotatus communis*	3	0.4	0	2	1	2
*Ochlerotatus excrucians*	1	0.1	0	0	1	1
*Ochlerotatus geniculatus*	2	0.3	0	0	2	1
*Ochlerotatus refiki*	1	0.1	0	0	1	1
*Ochlerotatus rusticus*	2	0.3	0	2	0	2
*Ochlerotatus sticticus*	16	2.1	1	13	2	7
Total (percentage)	775 (100 %)		51 (6.6 %)	572 (73.8 %)	152 (19.6)

**Table 2:** Frequency and percentage for each host species differentiated for three host-feeding groups (birds, non-human mammals and humans)

Host species	No. of mosquito specimens	Percentage of all collected specimens
Birds		
*Anas platyrhynchus*	1	0.1
*Corvus corone*	1	0.1
*Cyanistes caeruleus*	4	0.5
*Delichon urbica*	2	0.3
*Emberiza citrinella*	2	0.3
*Erithacus rubecula*	1	0.1
*Passer domesticus*	3	0.4
*Sylvia atricapilla*	3	0.4
*Sylvia communis*	2	0.3
*Turdus merula*	25	3.2
*Turdus philomelos*	7	0.9
Total	51	6.6
Humans		
*Homo sapiens*	152	19.6
Total	152	19.6
Non-human mammals		
*Bos taurus*	101	13.0
*Canis lupus familiaris*	1	0.1
*Capra hircus*	1	0.1
*Capreolus capreolus*	258	33.3
*Castor fiber*	2	0.3
*Cervus elaphus*	9	1.2
*Equus caballus*	6	0.8
*Erinaceus europaeus*	1	0.1
*Felis catus*	3	0.4
*Lama pacos*	1	0.1
*Lepus europaeus*	26	3.4
*Microtus guentheri*	2	0.3
*Myocastor coypus*	1	0.1
*Myodes glareolus*	1	0.1
*Myotis* spp.	2	0.3
*Oryctolagus cuniculus*	9	1.2
*Ovis aries*	28	3.6
*Rattus norvegicus*	1	0.1
*Sus scrofa*	116	15.0
*Vulpes vulpes*	3	0.4
Total	572	73.8

Non-human mammals dominated the host species (572 specimens, 73.8 % of all mosquito specimens), followed by humans (152, 19.6 %) and birds (51, 6.6 %) (Fig. 2, Table 2). Common hosts were roe deer (*Capreolus capreolus*; 258 mosquito specimens, 33.3 % of all mosquito specimens, 65 % of all mosquito species), humans (*H. sapiens*; 152, 19.6 %, 90 %), cattle (*Bos taurus*; 101, 13.0 %, 60 %), and wild boar (*Sus scrofa*; 116, 15.0 %, 50 %).

**Fig. 3:**
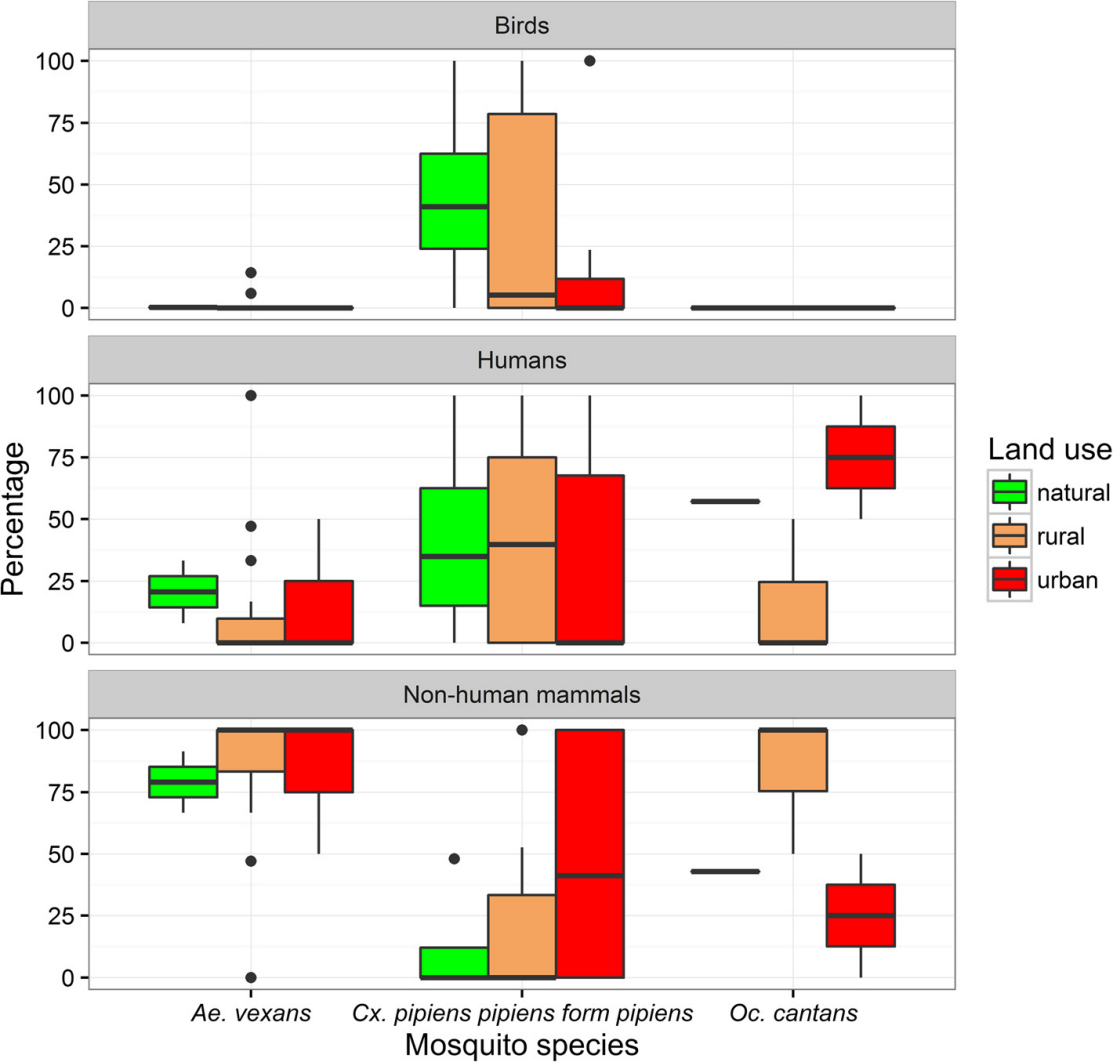
Percentage of each host group for the three most frequent mosquito species differentiated for three classes of land use at each trapping site

The surrounding land use did not have a statistical significant impact on the host-feeding patterns of the three most frequent species (*Ae. vexans*, *Cx. pipiens pipiens* form *pipiens* and *Oc. cantans*) (Kruskal-Wallis tests, Additional file 2: Table S2; Fig. 3). Only for the species *Oc. cantans*, the data indicated a higher percentage of humans in the urban areas and a higher percentage of non-human mammals in the rural areas, but these differences were statistically not significant. The same applies to the comparison of the host-feeding patterns between the early and late sampling period, for which also no statistically significant differences were found (chi-square tests; *Ae. vexans*: *χ*^*2*^ = 5.8, *df* = 2, *P* = 0.05502; *Cx. pipiens pipiens* form *pipiens*: *χ*^*2*^ = 0.40438, *df* = 2, *P* = 0.8169; and *Oc. cantans*: *χ*^*2*^ = 0.0033981, *df* = 1, *P* = 0.9535; Table 3). In addition, multiple chi-square tests indicated a significantly higher number of detections for mosquitoes with blood-meals from birds *vs* blood-meals from mammals for gravid traps compared against EVS and sweep nets (chi-square tests; gravid trap against EVS: *χ*^*2*^ = 88.107, *df* = 1, *adjusted P* < 0.0001; gravid trap against sweep net: *χ*^*2*^ = 29.518, *df* = 1, *adjusted P* < 0.0001), but no statistical differences between the other pairs of trapping methods (Additional file 3: Table S3).

**Table 3:** Frequency and percentage of the three most frequent mosquito species differentiated for host-feeding groups and sampling period [early (January to June) and late (July to December)]

Mosquito species	No. of mosquito specimens	Percentage of all collected specimens per species and sampling period	Host-feeding group	Sampling period
*Ae. vexans*	1	1.5	Birds	Early
*Ae. vexans*	1	1.5	Humans	Early
*Ae. vexans*	63	96.9	Non-human mammals	Early
*Ae. vexans*	3	1.0	Birds	Late
*Ae. vexans*	33	11.1	Humans	Late
*Ae. vexans*	262	87.9	Non-human mammals	Late
*Cx. pipiens pipiens* form *pipiens*	3	21.4	Birds	Early
*Cx. pipiens pipiens* form *pipiens*	5	35.7	Humans	Early
*Cx. pipiens pipiens* form *pipiens*	6	42.9	Non-human mammals	Early
*Cx. pipiens pipiens* form *pipiens*	25	29.1	Birds	Late
*Cx. pipiens pipiens* form *pipiens*	30	34.9	Humans	Late
*Cx. pipiens pipiens* form *pipiens*	31	36.0	Non-human mammals	Late
*Oc. cantans*	0	0	Birds	Early
*Oc. cantans*	19	27.1	Humans	Early
*Oc. cantans*	51	72.9	Non-human mammals	Early
*Oc. cantans*	0	0	Birds	Late
*Oc. cantans*	7	24.1	Humans	Late
*Oc. cantans*	22	75.9	Non-human mammals	Late

**Fig. 4:**
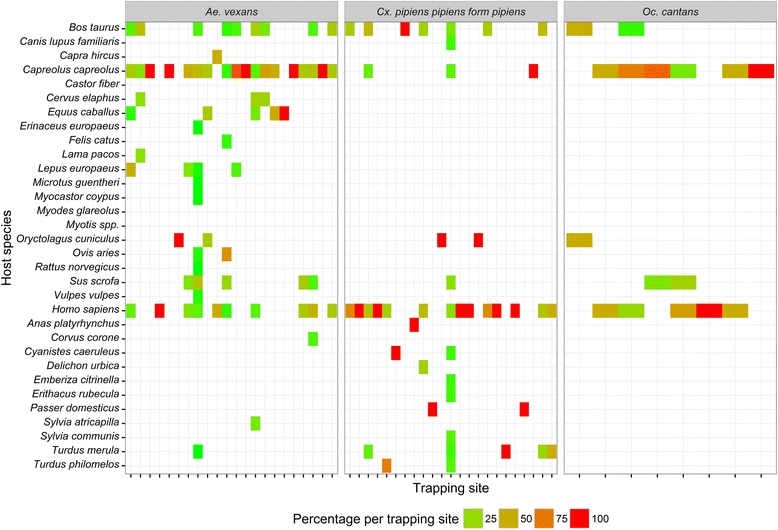
Percentage of each detected host species per trapping site for the three most frequently collected mosquito species

C-score null model tests demonstrated that the host species of the collected mosquito species had an aggregated pattern (Table 4, Fig. 2), indicating that most mosquito species shared at least one host species (e.g. 90 % of all mosquito species fed on humans). The same was observed for the three most frequent species (*Ae. vexans*, *Cx. pipiens pipiens* form *pipiens*, and *Oc. cantans*), which had an aggregated pattern for the host species compared between the trapping sites (Table 4). For *Ae. vexans*, roe deer was a common host, humans were frequently detected for *Cx. pipiens pipiens* form *pipiens*, and both, roe deer and humans, were common hosts of *Oc. cantans* (Fig. 4).

**Table 4:** Host-feeding patterns. Group indicates the dataset for the respective analysis. Values of the estimated C-score are the values calculated from the data, and Mean ± Variance are the results from the simulations. The values of *P* < exp and *P* > exp indicate the probability that the C-score value is significantly smaller (indicating aggregated pattern) or larger (segregated pattern) than that expected by random, with a *P*-value < 0.05 indicating statistical significance. When none of the *P*-values is below the threshold of 0.05 the pattern is random. Column “pattern” indicates the interpretation of the pattern

Group	C-score	Mean ± Variance	*P* < exp	*P* > exp	Pattern
Between all mosquito species	3.508	11.474 ± 0.199	<0.001	1	Aggregated
*Aedes vexans* between trapping sites	1.143	3.198 ± 0.069	<0.001	1	Aggregated
*Culex pipiens pipiens* form *pipiens* between trapping sites	1.225	2.838 ± 0.019	<0.001	1	Aggregated
*Ochlerotatus cantans* between trapping sites	1.143	3.534 ± 0.045	<0.001	1	Aggregated

**Fig. 5:**
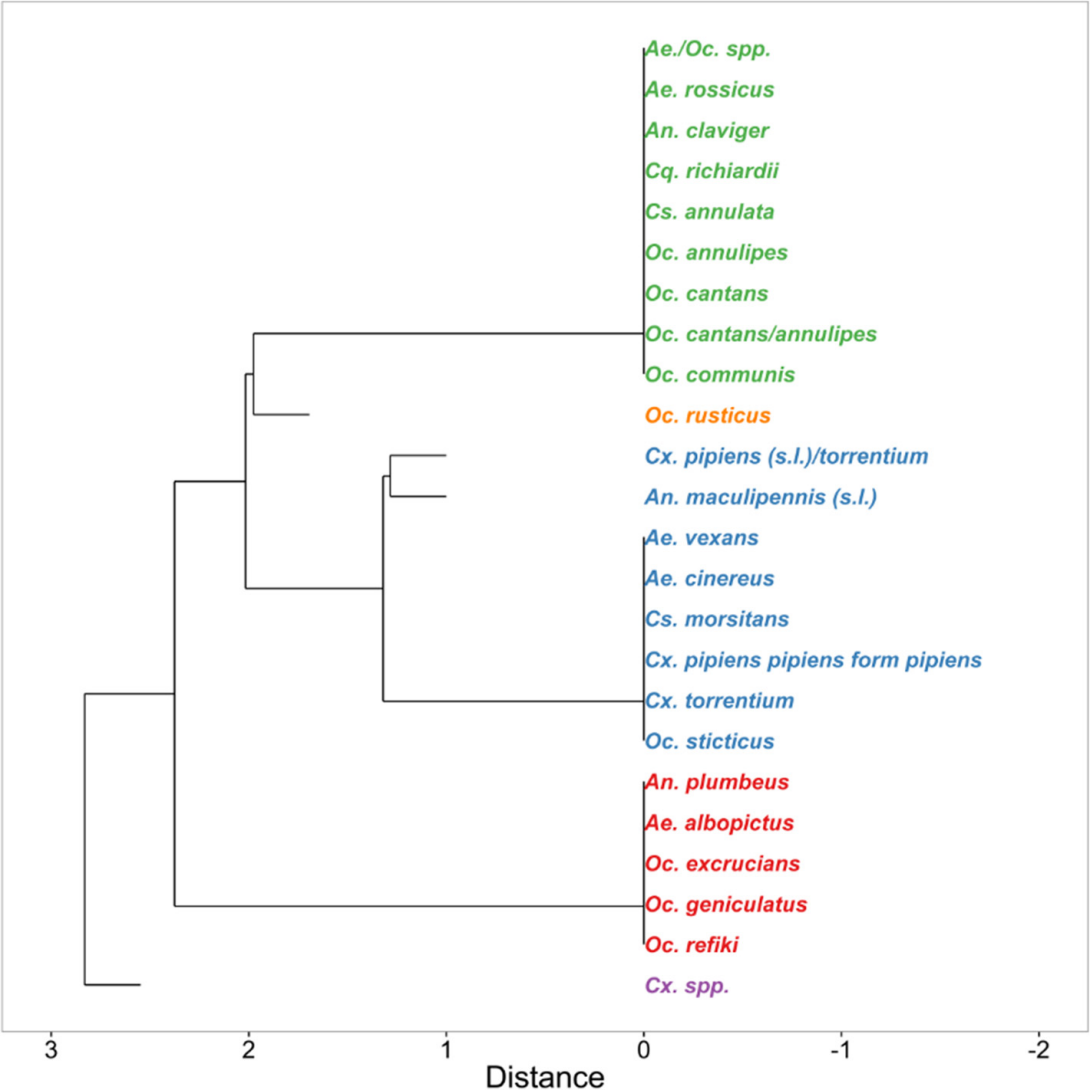
Dendrogram of the presence-absence dataset of host groups (birds, non-human mammals and humans) for each mosquito species (normalized presence-absence dataset) based on Jaccard’s dissimilarity index and average agglomeration method. Each colour indicates a host-feeding group of mosquito species with a unique combination of host groups: green, non-human mammals and humans; orange, non-human mammals; blue, birds, non-human mammals and humans; red, humans; purple, birds

**Fig. 6:**
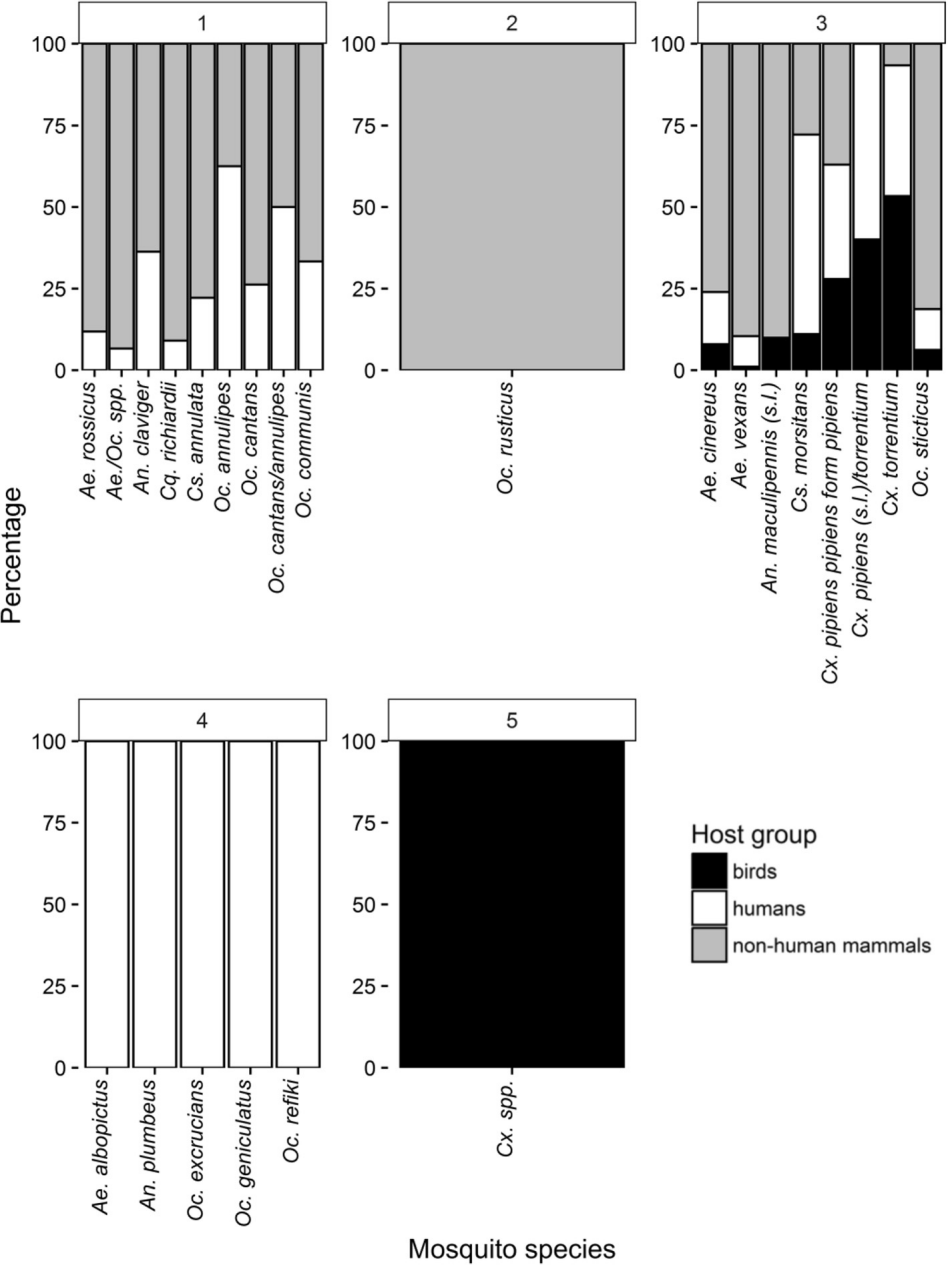
Percentage of host groups for each mosquito species aggregated in the host-feeding groups identified with cluster analysis (Fig. 5)

Five different host-feeding groups were identified (Figs. 5 and 6). Each group consists of mosquito species, which exclusively feed on one combination of host-feeding groups. Group 3 included all mosquito species with host species from all three host groups (birds, non-human mammals and humans), while all mosquito species in Group 1 feed only on non-human mammals and humans. Groups 2, 4 and 5 comprise mosquito species with a single host group per species since only one or two specimens of these mosquito species were collected.

**Fig. 7:**
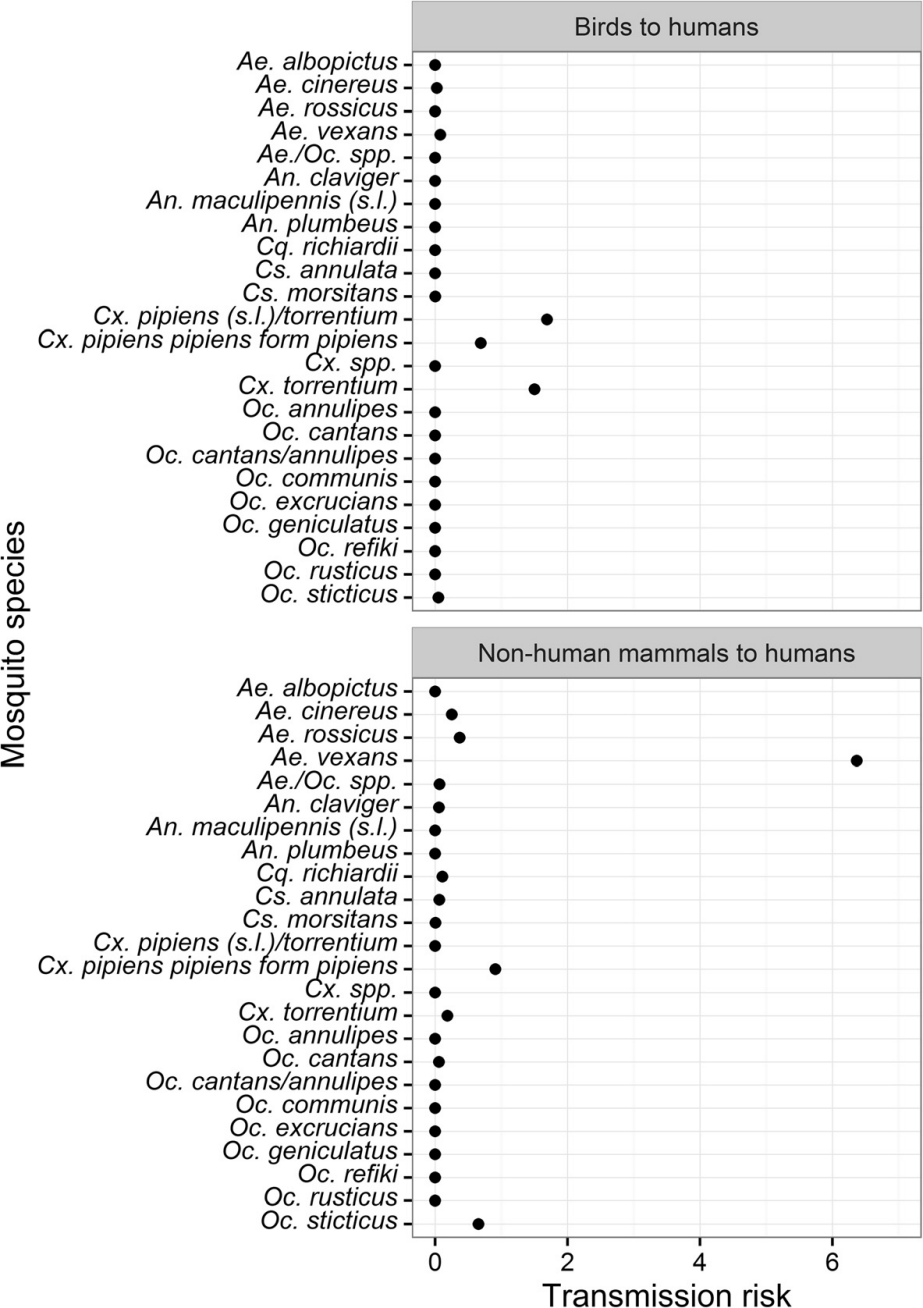
Estimates for the pathogen transmission risk for each mosquito species differentiated for a transmission between (i) birds and humans and (ii) non-human mammals and humans

The calculation of the potential transmission risks for each mosquito species demonstrated that *Cx. pipiens pipiens* form *pipiens* and *Cx. torrentium* collectively are potential vectors for pathogens transmitted from birds to humans, whereas *Ae. vexans* and *Cx. pipiens pipiens* form *pipiens* are potential vectors of pathogens transmitted from non-human mammals to humans (Fig. 7). Furthermore, to a slightly lesser extent, three species, *Ae. cinereus*, *Ae, rossicus* and *Oc. sticticus*, might be considered to play a potential role in the transmission of pathogens from non-human mammals to humans.

## Discussion

The host-feeding behaviour is an important parameter influencing the vector capacity of mosquito species [9]. The present study indicated an aggregated feeding pattern, which means that most of the mosquito species collected in Germany shared one or more host species [12]. Although we detected more than 32 host species, there was a distinctly higher frequency of mammals and especially roe deer, human, cattle and wild boar. There are at least two hypotheses, which could explain this general pattern. One explanation is a similar host species preference of all mosquito species. For example, all species except *An. maculipennis* (*s.l*.) and *Oc. rusticus* fed on humans, which is in agreement with similar studies from Switzerland or the USA [17, 37]. Another explanation would be that the host selection is based on the defensive behaviour or relative abundance in the community of potential hosts. An aggregated host-feeding pattern was also found between different trapping sites for the three most frequent species *Ae. vexans*, *Cx. pipiens pipiens* form *pipiens* and *Oc. cantans*. Thus, our study supports the conclusion by Chaves et al. [12] that specialization in host-feeding selection could not be found for adult mosquitoes. In the context of pathogen transmission, this result indicates that host-feeding patterns and thereby the risk of pathogen transmission is probably more strongly influenced by the host availability than by species-specific host choices.

Although there was a strong overlap between the host-feeding patterns of the different mosquito species, five host-feeding groups with the same combination of three host groups (birds, non-human mammals and humans) were identified. Group 1 included all mosquito species with exclusively feed on mammalian hosts including humans making them potential vectors for pathogens transmitted between mammals, e.g. BATV or filarial parasites. In contrast, all three host groups (birds, non-human mammals and humans) were detected for the mosquito species in Group 3, which predestine them as potential bridge vectors for both, pathogens only transmitted between mammals or for pathogens transmitted between mammals and birds, e.g. WNV and USUV [38]. Due to the low number of specimens, only a single host group was detected for several species (Group 2, 4 and 5), which probably does not reflect the complete host range and therefore is not further discussed here.

While the host-feeding patterns in the Group 1 match with the general classification found in the literature, the host range for some mosquito species for Group 3 indicated substantial differences. For example, *Cx. pipiens pipiens* form *pipiens*, *Cx. torrentium* and *Culiseta morsitans* are commonly classified as predominantly ornithophilic species [20, 39–41]. This leads to a categorisation as enzootic vectors for pathogens that circulate in bird populations, e.g. SINV [42] or WNV [38]. However, in the present study, blood meals from all three host-feeding groups were detected with significant amounts. Different publications on the feeding patterns of these species indicate no exclusive ornithophily [43] and even equivalent mammal and bird feeding was observed in the field [44]. The host-feeding patterns in combination with a wide distribution and abundance of the species [24, 45] confirms the potential of *Cx. pipiens pipiens* form *pipiens* or *Cx. torrentium* as bridge vectors for arboviruses [46, 47] such as USUV, already circulating in southern Germany [48]. Furthermore, *Cx. pipiens pipiens* form *pipiens* must be also considered as a vector of pathogens transmitted from non-human mammals to humans, e.g. Kronefeld et al. [6] detected the filarial nematode *D. immitis* in pools of *Cx. pipiens* (*s.l*.)/*torrentium* specimens.

Nevertheless, the identified host-feeding groups were solely based on the feeding patterns for the three differentiated host group, but the relative proportions of each group were not the same for the different mosquito species within the same group. This results in a different assessment of their vector capacity, e.g. although all three host-feeding groups (birds, non-human mammals and humans) were identified for *Ae. vexans*, the overwhelming majority of host species were mammals, which is in concordance with other studies [49, 50]. Thus, due to the host-feeding pattern and the high abundance in the sampling sites, *Ae. vexans* has to be considered as an important potential vector of pathogens from non-human mammals to humans (e.g. BATV), but not from birds to humans. This matches the classification by Medlock et al. [8], who evaluated *Ae. vexans* as being potentially involved in WNV transmission cycles, but not as an important bridge vector. A similiar transmission risk assesment probably applies to the species *Ae. cinereus* and *Oc. sticticus*, which are generally found or considered to be predominantly mammalophilic [17, 20, 40, 41].

Our data did not indicate any statistically significant spatial-temporal variation of the host-feeding patterns. However, as reviewed by Takken & Verhulst [11], many mosquito species exhibit a high degree of plasticity and therefore, host-feeding patterns may only reflect host availability. Future studies should especially focus on the spatial-temporal variation of host-feeding patterns in combination with studies on the species composition of the host community, e.g. changes in the host-feeding pattern of *Cx. pipiens* (*s.l*.) in response to the seasonality of host abundance, which significantly affects the pathogen epidemiology [51, 52]. Furthermore, the present study predominantly used blood-fed mosquitoes collected as by-catch within a nationwide mosquito and pathogen surveillance program. However, a higher specificity for gravid traps for mosquitoes with blood-meals from birds against blood-meals from mammals compared to EVS traps and sweep nets suggest that the study results might be slightly biased by the usage of different trapping methods. This is in agreement with other studies, demonstrating differences in the trapping efficacy and specificity of adult mosquitoes between diverse sampling devices [53]. Therefore, a standardized data collection with different methods at each trapping site might give a more comparable and complete picture of the host-feeding patterns of mosquitoes.

## Conclusions

Studies on host-feeding patterns of mosquitoes are a largely neglected field of research in Germany. This study provides a first, detailed insight into the host-feeding patterns of mosquitoes in this country. The species had a strong overlap for few host species, indicating that vector capacity is either very similar between different mosquito species or is highly dependent on the composition of the host community. Therefore, the risk of pathogen transmission is probably more strongly influenced by the host availability than by host-specific choices. Nevertheless, besides this overall aggregated host-feeding pattern, two different host-feeding groups were identified: (i) mosquito species feeding exclusively on non-human mammals and humans, which predestine these species as potential vectors for pathogens transmitted between mammals or (ii) mosquito species feeding on all three host-species groups (birds, non-human mammals and humans), which makes them bridge vectors for both, pathogens only transmitted between mammals and for pathogens transmitted between mammals and birds. *Culex pipiens pipiens* form *pipiens*/*torrentium* and *Ae. vexans* are widely distributed and in combination with their host-feeding patterns are probably relevant vector species for pathogens transmitted from birds to humans and non-human mammals to humans, respectively. Significant differences of the detected host-feeding patterns compared to general assumptions in the literature were detected for some of the species. For example, *Cs. morsitans* is commonly classified as predominantly ornithophilic species, but in our study, blood meals from birds, non-human mammals and humans were detected with significant amounts, indicating a potential role as both, enzootic and bridge vector for pathogens. Therefore, such kind of studies is urgently needed to further assess the vector capacity of the different species and the pathogen ecology.

## Abbreviations

Ap, percentage abundance of mosquitoes; BATV, Batai virus; Cv, measurement of the vector competence estimated as the proportion of bites from infected mosquitoes that transmit the virus; EVS, Heavy Duty Encephalitis Vector Survey trap; Fa, fraction of blood meals taken from avian hosts; Fh, fraction of blood meals taken from human hosts; Fm, fraction of blood meals taken from non-human mammals; PCR, polymerase chain reaction; SINV, Sindbis virus; USUV, Usutu virus; WNV, West Nile virus.

## Additional files

Additional file 1: Table S1. Trapping site information. (DOCX 19 kb) Additional file 2: Table S2. Kruskal-Wallis tests on the differences of the percentages of detected birds, non-human mammals and humans between the three land use classes (natural, rural and urban) for the three most frequent mosquito species (Fig. 3). (DOCX 14 kb) Additional file 3: Table S3. Chi-square tests on the differences in the frequencies of detected mammalian or avian hosts among all pairs of used trapping methods with adjusted *P*-values for multiple comparisons. (DOCX 13 kb)

## References

[1] Zeller H, Marrama L, Sudre B, Bortel WV, Warns-Petit E (2013). Mosquito-borne disease surveillance by the European Centre for Disease Prevention and Control. Clin Microbiol Infect.

[2] Jöst H, Bialonski A, Storch V, Günther S, Becker N, Schmidt-Chanasit J (2010). Isolation and phylogenetic analysis of Sindbis viruses from mosquitoes in Germany. J Clin Microbiol.

[3] Jöst H, Bialonski A, Schmetz C, Günther S, Becker N, Schmidt-Chanasit J (2011). Isolation and phylogenetic analysis of Batai virus, Germany. Am J Trop Med Hyg.

[4] Jöst H, Bialonski A, Maus D, Sambri V, Eiden M, Groschup MH (2011). Isolation of Usutu virus in Germany. Am J Trop Med Hyg.

[5] Czajka C, Becker N, Jöst H, Poppert S, Schmidt-Chanasit J, Krüger A, Tannich E (2014). Stable transmission of *Dirofilaria repens* nematodes, northern Germany. Emerg Infect Dis.

[6] Kronefeld M, Kampen H, Sassnau R, Werner D (2014). Molecular detection of *Dirofilaria immitis,* Dirofilaria repens and Setaria tundra in mosquitoes from Germany. Parasit Vectors.

[7] Melaun C, Zotzmann S, Santaella VG, Werblow A, Zumkowski-Xylander H, Kraiczy P, Klimpel S. Occurrence of *Borrelia burgdorferi* s.l. in different genera of mosquitoes (Culicidae) in Central Europe. Ticks Tick Borne Dis. 2015; doi:10.1016/j.ttbdis.2015.10.018. 10.1016/j.ttbdis.2015.10.018 26631488

[8] Medlock JM, Snow KR, Leach S (2005). Potential transmission of West Nile virus in the British Isles: an ecological review of candidate mosquito bridge vectors. Med Vet Entomol.

[9] Kramer LD, Ciota AT (2015). Dissecting vectorial capacity for mosquito-borne viruses. Curr Opin Virol.

[10] Ayres CFJ. Identification of Zika virus vectors and implications for control. Lancet Infect Dis. 2016; doi:10.1016/S1473-3099(16)00073-6. 10.1016/S1473-3099(16)00073-6 26852727

[11] Takken W, Verhulst NO (2013). Host preferences of blood-feeding mosquitoes. Annu Rev Entomol.

[12] Chaves LF, Harrington LC, Keogh CL, Nguyen AM, Kitron UD (2010). Blood feeding patterns of mosquitoes: random or structured?. Front Zool.

[13] Burkett-Cadena ND, Graham SP, Hassan HK, Guyer C, Eubanks MD, Katholi CR, Unnasch TR (2008). Blood feeding patterns of potential arbovirus vectors of the genus *Culex* targeting ectothermic hosts. Am J Trop Med Hyg.

[14] Alcaide M, Rico C, Ruiz S, Soriguer R, Muñoz J, Figuerola J (2009). Disentangling vector-borne transmission networks: a universal DNA barcoding method to identify vertebrate hosts from arthropod bloodmeals. PLoS One.

[15] Muñoz J, Ruiz S, Soriguer R, Alcaide M, Viana DS, Roiz D (2012). Feeding patterns of potential West Nile virus vectors in south-west Spain. PLoS One.

[16] la Martínez-de Puente J, Muñoz J, Capelli G, Montarsi F, Soriguer R, Arnoldi D, Rizzoli A (2015). Avian malaria parasites in the last supper: identifying encounters between parasites and the invasive Asian mosquito tiger and native mosquito species in Italy. Malar J.

[17] Schönenberger AC, Wagner S, Tuten HC, Schaffner F, Torgerson P, Furrer S (2015). Host preferences in host-seeking and blood-fed mosquitoes in Switzerland. Med Vet Entomol.

[18] Wesenberg-Lund C (1943). Biologie der Süsswasserinsekten.

[19] Martini E (1952). Lehrbuch der medizinischen Entomologie.

[20] Becker N, Krüger A, Kuhn C, Plenge-Bönig A, Thomas SM, Schmidt-Chanasit J, Tannich E (2014). Mosquitoes as vectors for exotic pathogens in Germany. Bundesgesundheitsbl.

[21] Reiter P (1983). A portable, battery-powered trap for collecting gravid *Culex* mosquitos. Mosq News.

[22] Mohrig W (1969). Die Culiciden Deutschlands.

[23] Becker N, Petric D, Zgomba M, Boase C, Madon M, Dahl C, Kaiser A (2010). Mosquitoes and Their Control.

[24] Rudolf M, Czajka C, Börstler J, Melaun C, Jöst H, von Thien H (2013). First nationwide surveillance of *Culex pipiens* complex and *Culex torrentium* mosquitoes demonstrated the presence of *Culex pipiens* biotype *pipiens/molestus* hybrids in Germany. PLoS One.

[25] Kitano T, Umetsu K, Tian W, Osawa M (2007). Two universal primer sets for species identification among vertebrates. Int J Legal Med.

[26] Kocher TD, Thomas WK, Meyer A, Edwards SV, Paabo S, Villablanca FX, Wilson AC (1989). Dynamics of mitochondrial DNA evolution in animals: amplification and sequencing with conserved primers. Proc Natl Acad Sci U S A.

[27] R Core Team. R: A language and environment for statistical computing. Vienna: R Foundation for Statistical Computing; 2015. https://www.R-project.org. Accessed 1 Apr 2016.

[28] Wickham H (2011). The split-apply-combine strategy for data analysis. J Stat Softw.

[29] Wickham H (2007). Reshaping data with the reshape package. J Stat Softw.

[30] Wickham H (2009). ggplot2: elegant graphics for data analysis.

[31] Auguie B. gridExtra: miscellaneous functions for “grid” graphics. R package version 2.0.0. 2015. http://CRAN.R-project.org/package=gridExtra. Accessed 1 Apr 2016.

[32] de Vries A, Ripley BD. ggdendro: create dendrograms and tree diagrams using ‘ggplot2′. R package version 0.1-17. 2015. http://CRAN.Rproject.org/package=ggdendro. Accessed 1 Apr 2016.

[33] Gotelli NJ, Hart EM, Ellison AM. EcoSimR-Alpha. 2015; doi:10.5281/zenodo.16522. Accessed 1 Apr 2016.

[34] Fife, D. Fifer. A collection of miscellaneous functions. R package version 1.0. 2014. http://CRAN.R-project.org/package=fifer. Accessed 1 Apr 2016.

[35] Oksanen J, Blanchet FG, Kindt R, Legendre P, Minchin PR, O’Hara RB, Simpson GL, Solymos P, Stevens MHH, Wagner, H. vegan: Community Ecology Package. R package version 2.3-2. 2015. http://CRAN.R-project.org/package=vegan. Accessed 1 Apr 2016.

[36] Leggewie M, Badusche M, Rudolf M, Jansen S, Börstler J, Krumkamp R (2016). *Culex pipiens* and *Culex torrentium* populations from Central Europe are susceptible to West Nile virus infection. One Health.

[37] Tuten HC (2011). Habitat characteristics of larval mosquitoes in zoos of South Carolina, USA. J Am Mosq Control Assoc.

[38] Nikolay B (2015). A review of West Nile and Usutu virus co-circulation in Europe: how much do transmission cycles overlap?. Trans R Soc Trop Med Hyg.

[39] Service MW (1994). The biology of *Culiseta morsitans* and *Culiseta litorea* (Diptera: Culicidae) in England. Bull Entomol Res.

[40] Schäfer ML, Lundström JO, Pfeffer M, Lundkvist E, Landin J (2004). Biological diversity versus risk for mosquito nuisance and disease transmission in constructed wetlands in southern Sweden. Med Vet Entomol.

[41] Bueno-Marí R, Jiménez-Peydró R (2011). Classification of Spanish mosquitoes in functional groups. J Am Mosq Control Assoc.

[42] Hesson JC, Verner-Carlsson J, Larsson A, Ahmed R, Lundkvist Å, Lundström JO (2015). *Culex torrentium* mosquito role as major enzootic vector defined by rate of Sindbis virus infection, Sweden, 2009. Emerg Infect Dis.

[43] Fritz ML, Walker ED, Miller JR, Severson DW, Dworkin I (2015). Divergent host preferences of above- and below-ground *Culex pipiens* mosquitoes and their hybrid offspring. Med Vet Entomol.

[44] Apperson CS, Hassan HK, Harrison BA, Savage HM, Aspen SE, Farajollahi A (2004). Host feeding patterns of established and potential mosquito vectors of West Nile virus in the eastern United States. Vector Borne Zoonotic Dis.

[45] Lühken R, Steinke S, Leggewie M, Tannich E, Krüger A, Becker S, Kiel E (2015). Physico-chemical characteristics of *Culex pipiens* sensu lato and *Culex torrentium* (Diptera: Culicidae) breeding sites in Germany. J Med Entomol.

[46] Kilpatrick AM, Kramer LD, Campbell SR, Alleyne EO, Dobson AP, Daszak P (2005). West Nile virus risk assessment and the bridge vector paradigm. Emerg Infect Dis.

[47] Reisen WK (2012). The contrasting bionomics of *Culex* mosquitoes in Western North America. J Am Mosq Control Assoc.

[48] Becker N, Jöst H, Ziegler U, Eiden M, Höper D, Emmerich P (2012). Epizootic emergence of Usutu virus in wild and captive birds in Germany. PLoS One.

[49] Balenghien T, Fouque F, Sabatier P, Bicout DJ (2006). Horse-, bird-, and human-seeking behavior and seasonal abundance of mosquitoes in a West Nile virus focus of southern France. J Med Entomol.

[50] Greenberg JA, DiMenna MA, Hanelt B, Hofkin BV (2011). Analysis of post-blood meal flight distances in mosquitoes utilizing zoo animal blood meals. J Vector Ecol.

[51] Kilpatrick AM, Kramer LD, Jones MJ, Marra PP, Daszak P (2006). West Nile virus epidemics in North America are driven by shifts in mosquito feeding behavior. PLoS Biol.

[52] Rizzoli A, Bolzoni L, Chadwick EA, Capelli G, Montarsi F, Grisenti M (2015). Understanding West Nile virus ecology in Europe: *Culex pipiens* host feeding preference in a hotspot of virus emergence. Parasit Vectors.

[53] Lühken R, Pfitzner W, Börstler J, Garms R, Huber K, Schork N (2014). Field evaluation of four widely used mosquito traps in Central Europe. Parasit Vectors.

