# Supplementary material for: Host-feeding patterns of mosquito species in Germany
Source: Parasit Vectors. 2016 Jun 3;9:318. doi: 10.1186/s13071-016-1597-z (PMC4893232; doi:10.1186/s13071-016-1597-z)
Supplement: Additional file 1: Table S1. — Trapping site information. (DOCX 19 kb) [file 13071_2016_1597_MOESM1_ESM.docx]

**Additional file 1: Table S1.** Trapping site information

| **ID** | **WGS84_x** | **WGS84_y** | **Federal state** | **Dominating land use** | **Blood-fed mosquito species** |
| --- | --- | --- | --- | --- | --- |
| 1 | 10.100377 | 53.484189 | Hamburg | rural | *Cs. annulata*, *Oc. cantans* |
| 2 | 8.935581 | 50.266103 | Hesse | rural | *Ae. cinereus*, *Ae. vexans*, *Cx. pipiens pipiens* form *pipiens*, *Oc. sticticus* |
| 3 | 10.1367 | 53.3633 | Lower Saxony | rural | *Cx. pipiens pipiens* form *pipiens* |
| 4 | 9.227096 | 53.985964 | Schleswig-Holstein | rural | *Cx. pipiens pipiens* form *pipiens* |
| 5 | 8.858907 | 54.134553 | Schleswig-Holstein | rural | *Cx. pipiens pipiens* form *pipiens* |
| 6 | 12.521975 | 47.857372 | Bavaria | rural | *Ae. cinereus*, *Ae. vexans*, *Cq. richiardii*, *Oc. sticticus* |
| 7 | 12.518842 | 47.902096 | Bavaria | rural | *Ae. vexans* |
| 8 | 12.441043 | 51.864845 | Saxony-Anhalt | rural | *Ae. cinereus*, *Ae. vexans*, *Ae.*/*Oc.* spp., *Cx. pipiens pipiens* form *pipiens* |
| 9 | 12.404037 | 51.842354 | Saxony-Anhalt | rural | *An. maculipennis (s.l.)* |
| 10 | 8.248843 | 49.569594 | Rhineland-Palatinate | rural | *Cx. pipiens pipiens* form *pipiens* |
| 11 | 9.989662 | 53.608752 | Hamburg | urban | *Oc. cantans* |
| 12 | 9.53021 | 47.621641 | Baden-Württemberg | rural | *Ae. vexans*, *Cs. annulata*, *Oc. communis* |
| 13 | 7.842779 | 48.014566 | Baden-Württemberg | urban | *Ae. albopictus*, *Cx. pipiens pipiens* form *pipiens* |
| 14 | 9.448586 | 54.690751 | Schleswig-Holstein | rural | *Ae. cinereus*, *An. claviger* |
| 15 | 9.448586 | 54.690751 | Schleswig-Holstein | rural | *Cq. richiardii* |
| 16 | 10.039365 | 53.633354 | Hamburg | urban | *Cx. pipiens pipiens* form *pipiens* |
| 17 | 11.156273 | 54.514206 | Schleswig-Holstein | rural | *Cx. pipiens pipiens* form *pipiens* |
| 18 | 8.456984 | 49.737625 | Hesse | rural | *Cq. richiardii* |
| 19 | 8.658622 | 50.58181 | Hesse | urban | *Ae. cinereus*, *Ae. vexans* |
| 20 | 12.742197 | 52.443156 | Brandenburg | rural | *Ae. cinereus*, *Cx. pipiens pipiens* form *pipiens*, *Oc. cantans* |
| 21 | 8.296038 | 49.307362 | Rhineland-Palatinate | rural | *Oc. cantans*, *Oc. rusticus* |
| 22 | 8.661142 | 49.415049 | Baden-Württemberg | urban | *Cx. pipiens pipiens* form *pipiens* |
| 23 | 8.635889 | 49.594521 | Baden-Württemberg | rural | *Cx. pipiens (s.l.)*/*torrentium* |
| 24 | 8.385994 | 49.150355 | Baden-Württemberg | rural | *Ae. cinereus*, *Ae. vexans* |
| 25 | 8.418172 | 49.824537 | Hesse | natural | *Ae. cinereus*, *Ae. rossicus*, *Ae. vexans*, *Ae.*/*Oc.* spp., *An. claviger*, *An. maculipennis (s.l.)*, *An. plumbeus*, *Cq. richiardii*, *Cs. annulata*, *Cx. pipiens pipiens* form *pipiens*, *Cx. torrentium*, *Oc. geniculatus*, *Oc. sticticus* |
| 26 | 8.983976 | 47.742575 | Baden-Württemberg | urban | *Ae. vexans*, *Cx. pipiens pipiens* form *pipiens* |
| 27 | 8.992208 | 47.730266 | Baden-Württemberg | urban | *Ae. vexans* |
| 28 | 8.490683 | 49.579683 | Hesse | rural | *Ae. vexans*, *An. maculipennis (s.l.)* |
| 29 | 12.35095024 | 51.25906816 | Saxony | natural | *Oc. cantans*, *Oc. excrucians*, *Oc. sticticus* |
| 30 | 8.148015 | 49.369099 | Rhineland-Palatinate | natural | *Cx. pipiens pipiens* form *pipiens* |
| 31 | 8.367823 | 49.67414 | Hesse | rural | *Ae. vexans*, *Oc. sticticus* |
| 32 | 8.363661 | 49.677196 | Hesse | rural | *Ae. vexans*, *Cs. annulata* |
| 33 | 14.249272 | 52.787516 | Brandenburg | rural | *Ae. vexans*, *An. claviger*, *An. maculipennis (s.l.)*, *Cs. annulata* |
| 34 | 14.219849 | 52.812713 | Brandenburg | rural | *Ae. vexans* |
| 35 | 10.03747 | 53.622347 | Hamburg | urban | *Cx. pipiens pipiens* form *pipiens*, *Oc. cantans* |
| 36 | 10.138718 | 53.687533 | Hamburg | rural | *Ae. vexans*, *Cs. annulata*, *Cs. morsitans*, *Cx. pipiens pipiens* form *pipiens*, *Cx. torrentium*, *Oc. annulipes*, *Oc. cantans*, *Oc. cantans*/*annulipes*, *Oc. communis*, *Oc. geniculatus* |
| 37 | 12.887575 | 48.77501 | Bavaria | rural | *Ae. vexans*, *An. maculipennis (s.l.)* |
| 38 | 10.083344 | 53.664433 | Hamburg | urban | *Cx. pipiens pipiens* form *pipiens*, *Cx.* spp., *Cx. torrentium* |
| 39 | 10.083344 | 53.664433 | Hamburg | urban | *Cx. torrentium* |
| 40 | 7.293696 | 53.027894 | Lower Saxony | rural | *An. maculipennis (s.l.)*, *Cs. annulata* |
| 41 | 13.332878 | 54.178653 | Mecklenburg-Vorpommern | rural | *Ae. cinereus*, *Ae. rossicus*, *Ae. vexans*, *Ae.*/*Oc.* spp., *Oc. cantans*, *Oc. refiki* |
| 42 | 13.329749 | 54.177991 | Mecklenburg-Vorpommern | rural | *Cq. richiardii* |
| 43 | 8.410604 | 49.294566 | Rhineland-Palatinate | rural | *Cx. pipiens pipiens* form *pipiens* |
| 44 | 8.399148 | 49.292601 | Rhineland-Palatinate | rural | *Cx. pipiens pipiens* form *pipiens* |
| 45 | 8.414734 | 49.189821 | Baden-Württemberg | rural | *Ae. vexans* |
| 46 | 12.919235 | 48.787025 | Bavaria | rural | Ae. cinereus, *Ae. vexans*, *An. claviger*, *An. maculipennis (s.l.)* |
| 47 | 12.919235 | 48.787025 | Bavaria | rural | *Ae. vexans*, *An. claviger*, *An. maculipennis (s.l.)* |
| 48 | 8.523737 | 49.260604 | Baden-Württemberg | natural | *Ae. vexans*, *An. claviger*, *Cs. annulata*, *Cx. pipiens pipiens* form *pipiens*, Cx. pipiens (s.l.)/*torrentium*, *Oc. annulipes* |
| 49 | 9.62795 | 53.612037 | Schleswig-Holstein | rural | *Cx. pipiens pipiens* form *pipiens* |
| 50 | 8.667714 | 49.54991 | Baden-Württemberg | urban | *Cs. annulata*, *Cx. pipiens pipiens* form *pipiens*, *Cx. pipiens* *(s.l.)*/*torrentium*, *Cx. torrentium* |
| 51 | 8.671717 | 49.52411 | Baden-Württemberg | natural | *Cx. pipiens pipiens* form *pipiens*, *Cx. pipiens (s.l.)*/*torrentium*, *Cx. torrentium* |
| 52 | 10.115469 | 53.720292 | Hamburg | rural | *An. claviger*, *An. maculipennis (s.l.)*, *Cs. annulata*, *Cx. torrentium* |
