# Supplementary material for: Host-feeding patterns of mosquito species in Germany
Source: Parasit Vectors. 2016 Jun 3;9:318. doi: 10.1186/s13071-016-1597-z (PMC4893232; doi:10.1186/s13071-016-1597-z)
Supplement: Additional file 2: Table S2. — Kruskal-Wallis tests on the differences of the percentages of detected birds, non-human mammals and humans between the three land use classes (natural, rural and urban) for the three most frequent mosquito species (Fig. 3). (DOCX 14 kb) [file 13071_2016_1597_MOESM2_ESM.docx]

**Additional file 2: Table S2.** Kruskal-Wallis tests on the differences of the percentages of detected birds, non-human mammals and humans between the three land use classes (natural, rural and urban) for the three most frequent mosquito species (Fig. 3)

| **Host-feeding group** | **Mosquito species** | ***χ^2^*** | ***df*** | ***P*** |
| --- | --- | --- | --- | --- |
| birds | *Ae. vexans* | 2.1045027 | 2 | 0.34915081 |
| birds | *Cx. pipiens pipiens* form *pipiens* | 2.0306766 | 2 | 0.36227985 |
| birds | *Oc. cantans* | NA | NA | NA |
| humans | *Ae. vexans* | 1.7289817 | 2 | 0.42126599 |
| humans | *Cx. pipiens pipiens* form *pipiens* | 0.5857806 | 2 | 0.746104 |
| humans | *Oc. cantans* | 4.6386076 | 2 | 0.09834203 |
| non-human mammals | *Ae. vexans* | 1.4048973 | 2 | 0.49537082 |
| non-human mammals | *Cx. pipiens pipiens* form *pipiens* | 2.0456048 | 2 | 0.35958582 |
| non-human mammals | *Oc. cantans* | 4.6386076 | 2 | 0.09834203 |
