# Supplementary material for: Host-feeding patterns of mosquito species in Germany
Source: Parasit Vectors. 2016 Jun 3;9:318. doi: 10.1186/s13071-016-1597-z (PMC4893232; doi:10.1186/s13071-016-1597-z)
Supplement: Additional file 3: Table S3. — Chi-square tests on the differences in the frequencies of detected mammalian or avian hosts among all pairs of used trapping methods with adjusted P-values for multiple comparisons. (DOCX 13 kb) [file 13071_2016_1597_MOESM3_ESM.docx]

**Additional file 3: Table S3** Chi-square tests on the differences in the frequencies of detected mammalian or avian hosts among all pairs of used trapping methods with adjusted *P*-values for multiple comparisons

| **Comparison** | ***χ^2^*** | ***df*** | ***adjusted P*** |
| --- | --- | --- | --- |
| BG vs. BG+CO2 | 1.76E-31 | 1 | 1 |
| BG vs. EVS | 5.21E-27 | 1 | 1 |
| BG vs. EVS+yeast | NA | NA | NA |
| BG vs. gravid trap | 0.72917 | 1 | 0.7382 |
| BG vs. human-bait collection | NA | NA | NA |
| BG vs. sweep net | 1.70E-30 | 1 | 1 |
| BG vs. hand held aspirator | 1.76E-30 | 1 | 1 |
| BG+CO2 vs. EVS | 5.7809 | 1 | 0.0821 |
| BG+CO2 vs. EVS+yeast | 2.27E-30 | 1 | 1 |
| BG+CO2 vs. gravid trap | 5.7847 | 1 | 0.0734 |
| BG+CO2 vs. human-bait collection | 1.76E-31 | 1 | 1 |
| BG+CO2 vs. sweep net | 5.279 | 1 | 0.0734 |
| BG+CO2 vs. hand held aspirator | 0.0060343 | 1 | 1 |
| EVS vs. EVS+yeast | 2.25E-27 | 1 | 1 |
| EVS vs. gravid trap | 88.107 | 1 | < 0.0001 |
| EVS vs. human-bait collection | 5.21E-27 | 1 | 1 |
| EVS vs. sweep net | 0.65844 | 1 | 1 |
| EVS vs. hand held aspirator | 0.49374 | 1 | 0.7382 |
| EVS+yeast vs. gravid trap | 6.64E-32 | 1 | 1 |
| EVS+yeast vs. human-bait collection | NA | NA | NA |
| EVS+yeast vs. sweep net | 7.37E-28 | 1 | 1 |
| EVS+yeast vs. hand held aspirator | 1.39E-30 | 1 | 1 |
| gravid trap vs. human-bait collection | 0.72917 | 1 | 0.7382 |
| gravid trap vs. sweep net | 29.518 | 1 | < 0.0001 |
| gravid trap vs. hand held aspirator | 5.2743 | 1 | 0.0734 |
| human-bait collection vs. sweep net | 1.70E-30 | 1 | 1 |
| human-bait collection vs. hand held aspirator | 1.76E-30 | 1 | 1 |
| sweep net vs. hand held aspirator | 1.4206 | 1 | 0.4817 |
